# Fusion of *ZMYND8* and *RELA* Genes in Acute Erythroid Leukemia

**DOI:** 10.1371/journal.pone.0063663

**Published:** 2013-05-07

**Authors:** Ioannis Panagopoulos, Francesca Micci, Jim Thorsen, Lisbeth Haugom, Jochen Buechner, Gitte Kerndrup, Anne Tierens, Bernward Zeller, Sverre Heim

**Affiliations:** 1 Section for Cancer Cytogenetics, Institute for Medical Informatics, The Norwegian Radium Hospital, Oslo University Hospital, Oslo, Norway; 2 Centre for Cancer Biomedicine, Faculty of Medicine, University of Oslo, Oslo, Norway; 3 Department of Pediatrics, University Hospital of North-Norway, Tromsø, Norway; 4 Department of Pediatrics, Oslo University Hospital, Oslo, Norway; 5 Department of Pathology, Vejle Hospital, Vejle, Denmark; 6 Department of Pathology, The Norwegian Radium Hospital, Oslo University Hospital, Oslo, Norway; 7 Faculty of Medicine, University of Oslo, Oslo, Norway; Université Paris-Diderot, France

## Abstract

Acute erythroid leukemia was diagnosed in a 4-month-old boy. Cytogenetic analysis of bone marrow (BM) cells showed a t(11;20)(p11;q11) translocation. RNA extracted from the BM was sequenced and analyzed for fusion transcripts using the software FusionMap. A *ZMYND8-RELA* fusion was ranked first. RT-PCR and direct sequencing verified the presence of an in frame *ZMYND8-RELA* chimeric transcript. Fluorescence in situ hybridization showed that the *ZMYND8-RELA* was located on the p12 band of der(11); therefore a cytogenetically invisible pericentric inversion in chromosome 11 must have taken place besides the translocation. The putative ZMYND8-RELA fusion protein contains the Zinc-PHD finger domain, a bromodomain, a PWWP domain, a MYND type of zinc finger of ZMYND8, and the entire RELA protein, indicating that it might act leukemogenically by influencing several cellular processes including the NF-kappa-B pathway.

## Introduction

Acute erythroid leukemia (AEL) is an uncommon (5%) type of acute myeloid leukemia (AML). AEL or AML FAB M6 is characterized by a predominantly erythroid cell proliferation in the bone marrow. According to the 2008 World Health Organization (WHO) classification [1] there are two subtypes: 1) The erythroleukemia subtype, which is the most common and is defined by the presence in the marrow of 50% or more erythroid precursors and 20% or more blasts in the nonerythroid component, and 2) The pure erythroid leukemia subtype in which more than 80% immature erythroblasts are seen in the marrow. AEL can overlap morphologically with other, clinically distinct myeloid malignancies such as, AML with myelodysplasia-related changes, and myelodysplastic syndrome (MDS) [2], [3], [4]. Also for this reason it is of interest to distinguish these entities genetically, if indeed genetically distinct they are.

The Mitelman database of chromosome aberrations in cancer (http://cgap.nci.nih.gov/Chromosomes/Mitelman; database last update February 18, 2013) lists 632 cases of AEL with abnormal karyotypes. The most frequent aberrations, which are found also in other AML subtypes, are structural and numerical changes of chromosomes 5 and 7 as well as trisomy 8. Fusion genes brought about by chromosomal translocations have also been reported in acute erythroid leukemias: *NPM1/MLF1* resulting from t(3;5)(q25;q35), *RPN1/PRDM16* from t(1;3)(p36;q21), *PCM1/JAK2* from t(8;9)(p22;p24), and *NUP98/HOXD13* from t(2;11)(q31;p15) (http://cgap.nci.nih.gov/Chromosomes/Mitelman; database last update February 18, 2013). Recently we described a chimeric *NFIA/CBFA2T3* gene in a 15-month-old boy with acute erythroid leukemia carrying the chromosomal translocation t(1;16)(p31;q22) [5], [6]. In the present study, we report the fusion of the genes *ZMYND8* and *RELA* in the bone marrow cells from an infant with erythroid leukemia. Infant leukemia is characterized by a high leukocyte count, hepatosplenomegaly and frequent central nervous system involvement [7]. The most common (cyto)genetic event in infant leukemias is rearrangement of the *MLL* gene in chromosome band 11q23 which, depending on the applied methodology, may be as high as 85% [7]. The chromosome translocations t(1;22)(p13;q13) which gives rise to the *RBM15-MKL1* fusion gene and t(7;12)(q36;p13) resulting in the formation of the *MNX1-ETV6* fusion or overexpression of the *MNX1* gene have also been exclusively reported in infant leukemias [8].

## Materials and Methods

### Ethics Statement

The study was approved by the regional ethics committee (Regional komité for medisinsk forskningsetikk Sør-Øst, Norge, http://helseforskning.etikkom.no), and written informed consent was obtained from the patient´s parents to publication of the case details.

### Case history

**Figure 1:**
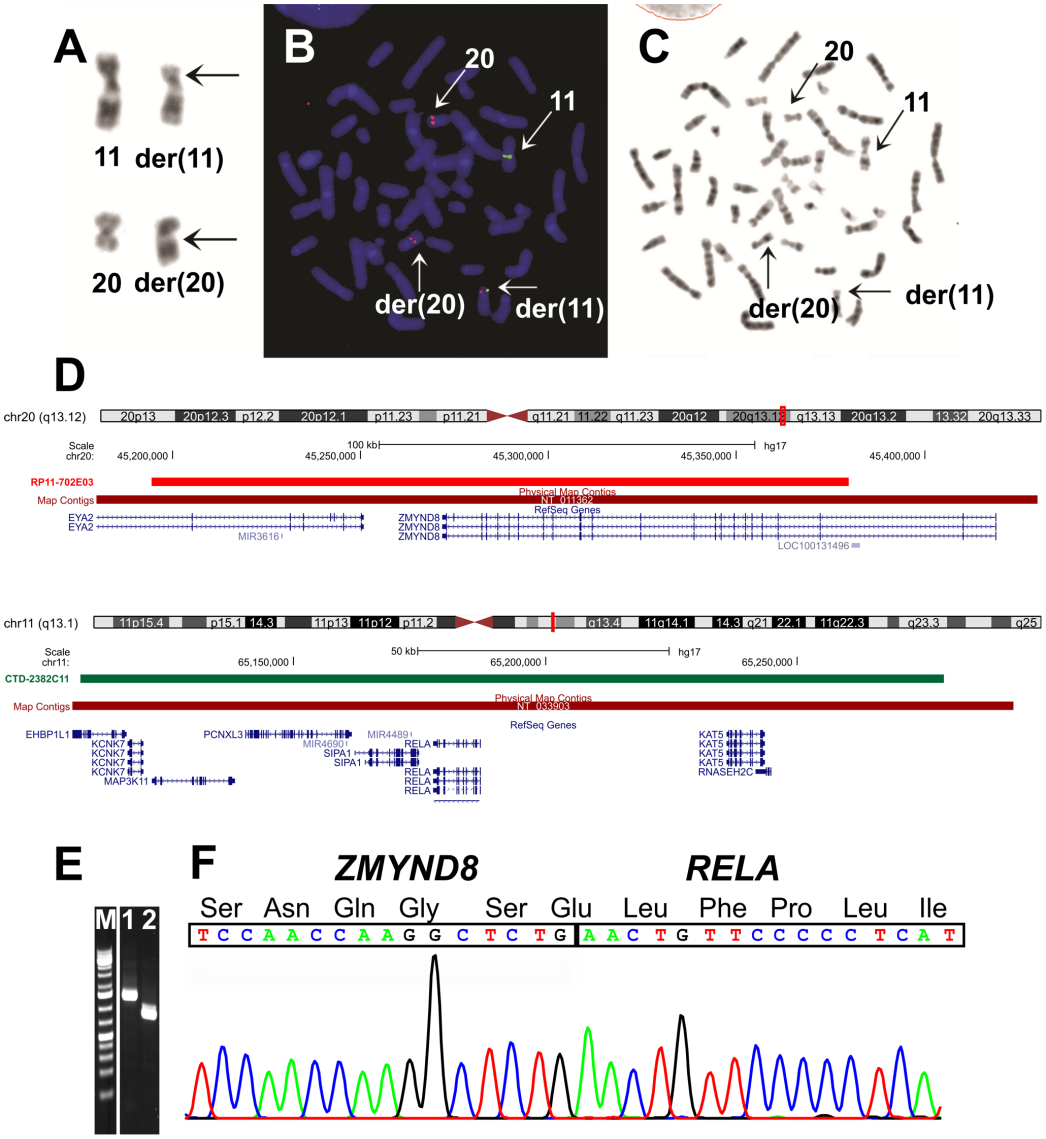
Cytogenetic, FISH, and PCR analyses of the erythroid leukemia. A) Partial G-banded karyotype showing what appeared to be chromosome aberrations der(11)t(11;20)(p11;q13) and der(20)t(11;20)(p11;q13) together with their corresponding normal homologues; breakpoint positions are indicated by arrows. B) FISH using BAC RP11-702E03 (red signal) from 20q13 containing the *ZMYND8* gene and BAC CTD-2382C11 (green signal) for *RELA*. A part of the probe RP11-702E03 for *ZMYND8* as well as the entire probe CTD-2382C11 for *RELA* had moved to band p12 of the derivative chromosome 11. The data suggest that the functional fusion gene is generated on the der(11). C) G-banding of the metaphase spread shown in (B). D) Mapping position of the RP11-702E03 on chromosome band 20q13 and CTD-2382C11 on chromosome band 11q13. E) Amplification of *ZMYND8-RELA* fusion cDNA fragments using ZMYND8-2683F and RELA-516R primer sets (lane 1) and ZMYND8-3079F and RELA-516R primers (lane 2). M, 1 Kb DNA ladder. F) Partial sequence chromatogram showing the junctions of the *ZMYND8-RELA* chimeric transcript and part of the in-frame coding protein.

A 4-month-old boy was referred to hospital with fatigue, irritability, regurgitation, and pallor for the last 3 weeks. On admission, the patient had high fever and hepatosplenomegaly, but otherwise normal physical findings. No congenital abnormalities or dysmorphic features were seen. Initial blood tests showed hemoglobin 6.7 g/dl, white blood cell count 24.9×10^9^/L, platelet count 116×10^9^/L, hemoglobin F 12%, and lactate dehydrogenase 8400 U/L. Peripheral blood smears demonstrated polychromasia and circulating dysplastic erythroblasts of all maturation stages, some metamyelocytes and myelocytes, but no myeloblasts. Examination of a bone marrow (BM) aspirate revealed erythroid hyperplasia and dysplasia with binucleated and multinucleated forms. The aspirate smear count displayed 4% myeloblasts, 38% proerythroblasts, 25% polychromatic erythroblasts, and 22% orthochromatic erythroblasts. Histological examination of the BM revealed high cellularity, dysplastic megakaryocytes, and slight fibrosis around sinusoids. The number of CD34+ cells in the biopsy was around 8%. Flow cytometric immunophenotyping of consecutive bone marrow aspirates showed a marked increase of CD45 negative erytroblasts, ranging from 30-60% of total cells. They expressed CD71, glycophorin, and CD36, but were negative for other cell lineage markers such as CD13, CD33, CD11b, T-and B-cell markers as well as HLA-DR antigens. The CD34+ cells comprised 1.5% of total cells, and were mainly CD34+ B-cell precursor cells. The CD34+ myeloid precursor cells displayed a normal expression of CD34, CD117, HLA-DR antigens, CD13, and CD33. Granulopoiesis revealed a normal maturation pattern as revealed by the expression of CD13 and CD11b. Cytogenetic analysis of BM cells showed the abnormal karyotype 46,XY,t(11;20)(p11;q11) in 8 out of 10 metaphases (Figure 1A and 1C). A myeloid malignancy, either myelodysplastic syndrome or an early phase of AML, possibly with an infection superimposed, was suspected. Prior to the cytogenetics, congenital dyserythropoietic anemia (CDA) was considered and ruled out. Slides of the patient´s bone marrow were also evaluated at the CDA Diagnostic Service and Research Laboratory, MRC Molecular Haematology Unit, Weatherall Institute of Molecular Medicine, Oxord, UK. The morphological appearance was not diagnostic for CDA. During an observational period the child's general condition deteriorated, and hemophagocytic lymphohistiocytosis (HLH) was assumed. Hemophagocytosis was present in the bone marrow. In addition, HLH was suggested due to a combination of clinical criteria (splenomegaly, fever) and the laboratory constellation with cytopenia, hypertriglyceridemia, hyperferritinemia, and neurological symptoms. No infectious agents were detected. Cytology of the cerebrospinal fluid and brain-MRI were normal. Treatment according to an HLH-protocol [9] was initiated. However, repeated BM investigations now demonstrated 100% cellularity and > 70% CD71+ erythroblasts, indicative of a transition to acute erythroid leukemia. The patient did not completely fullfill the criteria “more than 80% erythroblasts” (M6b), nor “more than 20% blasts in the nonerythroid compartment” (M6a) as required by the WHO definition of pure erythroid leukemia [1], which might simply be due to the fact that treatment was started before he reached the appropriate counts. Concurrently, cytogenetic analysis showed clonal evolution to a more complex karyotype: 48,XY,+7,t(11;20)(p11;q11),+17[4]/48,XY,+del(7)(p11)t(11;20)(p11;q11),+17[9]/46,XY[2]. Therefore, HLH-treatment was stopped and AML treatment according to the current Nordic AML protocol begun instead [10]. Morphological bone marrow remission was achieved after the first of two induction courses. After the first consolidation course, the patient was transplanted in cytogenetic remission from a matched unrelated donor after conditioning with busulfan, cyclophosphamide, and melphalan [11]. He is now in complete remission 8 months after the transplantation.

### Molecular genetic analyses

Four μg of total RNA extracted from the patient´s bone marrow at the time of diagnosis were sent for high-throughput paired-end RNA-sequencing to the Norwegian Sequencing Centre at Ullevål Hospital (http://www.sequencing.uio.no/). The Illumina software pipeline was used to process image data into raw sequencing data and only sequence reads marked as “passed filtering” were used in the downstream data analysis. A total of 100 million reads were obtained. The FASTQC software was used for quality control of the raw sequence data (http://www.bioinformatics.babraham.ac.uk/projects/fastqc/). The software FusionMap was used for the discovery of fusion transcripts [12] (release date 2012-04-16) together with the pre-built Human B37 and RefGene from the FusionMap website (http://www.omicsoft.com/fusionmap/).

One μg of total RNA was reverse-transcribed in a 20 µL reaction volume using iScript Advanced cDNA Synthesis Kit for RT-qPCR according to the manufacturer's instructions (Biorad). The cDNA was diluted to 50 µL and 2 µL were used as templates in subsequent PCR assays. The 25 µL PCR volume contained 12.5 µL of Premix Taq (TaKaRa), 1 µL of diluted cDNA, and 0.2 µM of each of the forward ZMYND8-2683F (TCCATGAGCACCCTTGTGTCCTCAG) and reverse RELA-516R (GCGCTGACTGATAGCCTGCTCCAG) primers or ZMYND8-3079F (AAGGAGGCCATCTTTTACTGCTGTTGG) and RELA-516R. The PCR was run on a C-1000 Thermal cycler (Biorad) with an initial denaturation at 94°C for 30 sec, followed by 35 cycles of 7 sec at 98°C, 2 min at 68°C, and a final extension for 5 min at 68°C. Four μL of the PCR products were stained with GelRed (Biotium), analyzed by electrophoresis through 1.0% agarose gel, and photographed. The remaining PCR products were purified using the Qiagen PCR purification kit (Qiagen) and direct sequencing was performed using the light run sequencing service of GATC Biotech (http://www.gatc-biotech.com/en/sanger-services/lightrun-sequencing.html). The BLAST software (http://www.ncbi.nlm.nih.gov/BLAST/) was used for computer analysis of sequence data.

### Fluorescence in situ hybridization (FISH) analyses

**Figure 2:**
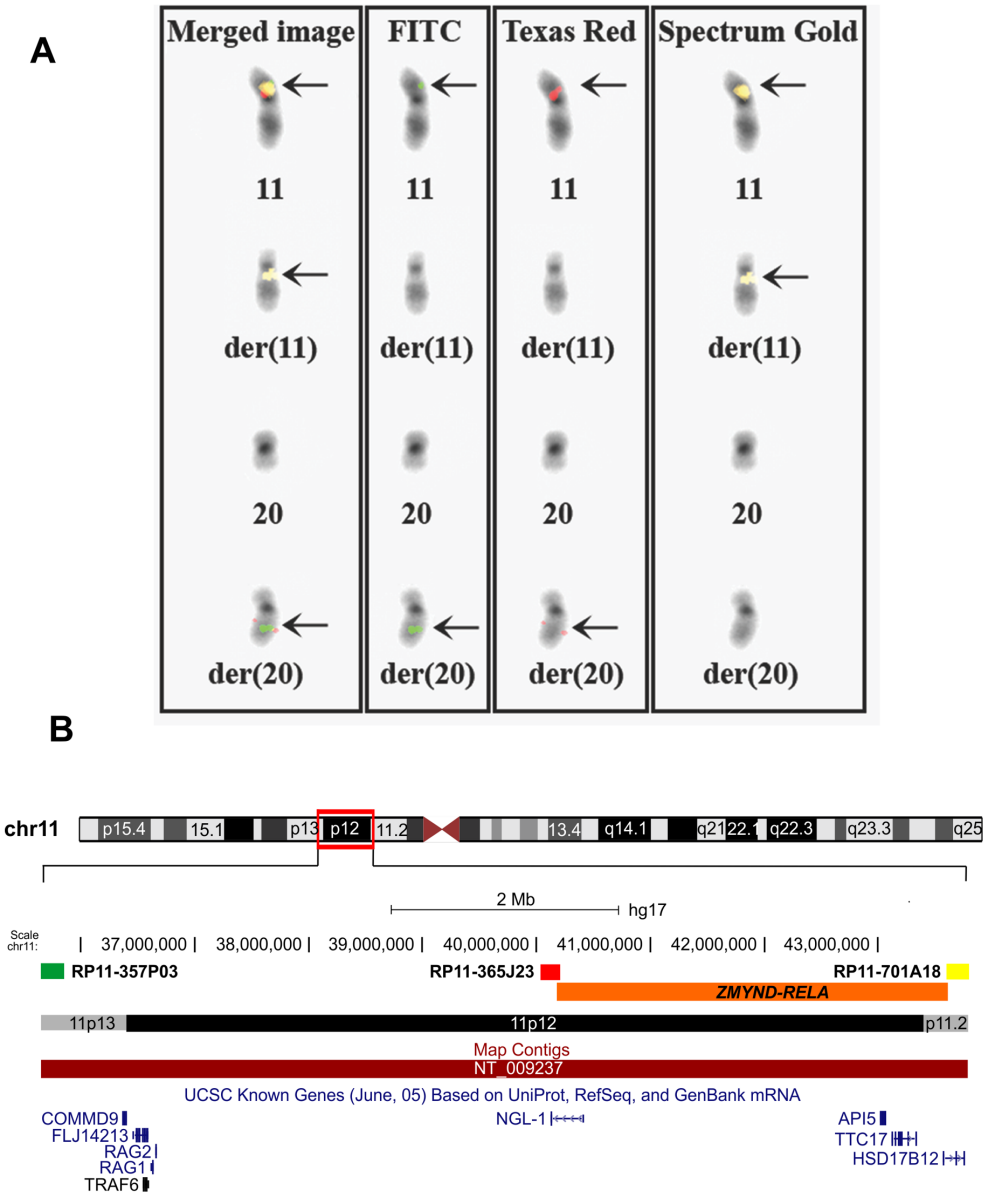
FISH using the BACs RP11-357P03 (FITC, green), RP11-365J23 (Texas Red, red) and RP11-701A18 (Spectrum Gold, yellow) for the localization of the fusion *ZMYND8-RELA* gene. A) The BACs RP11-357P03 (FITC) and RP11-365J23 (Texas Red) have moved to the q arm of der(20), whereas the probe RP11-701A18 (Spectrum Gold) had moved to the q arm of the derivative chromosome 11. B) Mapping position of the RP11-357P03, RP11-365J23 and RP11-701A18 in chromosome band 11p12. The localization of the fusion *ZMYND8-RELA* gene is between BACs RP11-365J23 and RP11-701A18.

The BAC probes, retrieved from the Human “32K” BAC Re-Array library (BACPAC Resources, http://bacpac.chori.org/home.htm), were: RP11-702E03 for *ZMYND8* (Figure 1D, Position: chr20:45194474-45379760; Band: 20q13.12; UCSC Genome Browser on Human May 2004 NCBI35/hg17 Assembly), CTD-2382C11 for *RELA* (Figure 1D, Position: chr11:65106839-65279190 on Human May 2004 NCBI35/hg17 Assembly) and RP11-357P03 and RP11-365J23 and RP11-701A18 for chromosome band 11p12 (Figure 2B). FISH was performed as described elsewhere [5], [6]

## Results and Discussion

Using FusionMap on the raw sequencing data obtained by the Norwegian Sequencing Centre, more than 1000 fusion genes were found. The *ZMYND8-RELA* fusion transcript was ranked first with 179 seed counts. Because of this and because of the translocation between chromosomes 11 and 20 detected by karyotyping (*ZMYND8* and *RELA* map to chromosome bands 20q13 and 11q13, respectively), we decided to investigate further the *ZMYND8-RELA* fusion transcript. No other fusions were examined. PCR and direct sequencing verified the presence of a *ZMYND8-RELA* chimeric transcript in which exon 21 of *ZMYND8*, from sub-band 20q13.12 (nt 3406 insequence with accession number NM_183047 version 1), was fused in frame to exon 2 of *RELA* from sub-band 11q13.1 (nt 148 in NM_021975 version 3) (Figure 1E and F). FISH showed that part of the probe RP11-702E03 for *ZMYND8* as well as the entire probe CTD-2382C11 for *RELA* had moved to band p12 of the derivative chromosome 11 (Figure 1B).The data suggested that the *ZMYND8-RELA* fusion gene was located on the p12 band of der(11). To further characterize the location of the *ZMYND8-RELA* fusion gene on the derivative chromosome 11, more chromosome 11-specific BAC clones from the Human “32K” BAC Re-Array library were used. We found that the BACs RP11-357P03 and RP11-365J23 had moved to the q arm of der(20), whereas the probe RP11-701A18 had moved to the q arm of the derivative chromosome 11 (Figure 2A). Taken together the FISH data suggest that the fusion *ZMYND8-RELA* gene is localized between BACs RP11-365J23 and RP11-701A18 (Figure 2B), and thus that a small pericentromeric inversion between bands 11p12 and 11q13 not detected with banding cytogenetics (G-banding had been used for karyotyping), had occurred.

**Figure 3:**
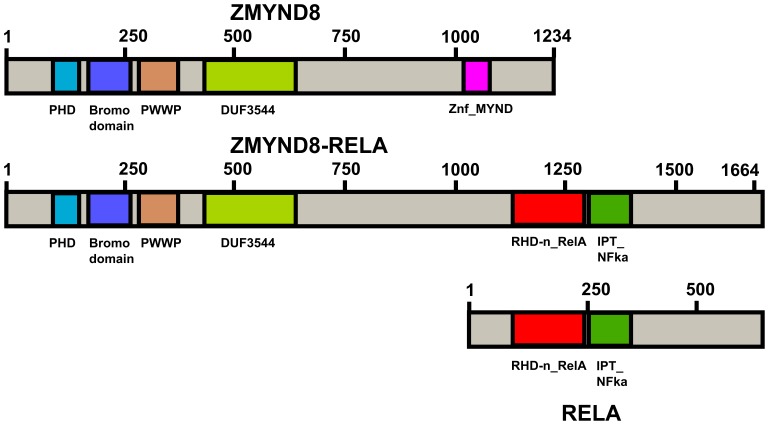
Diagram showing the domains of ZMYND8, RELA and the fusion ZMYND8-RELA protein.

The ZMYND8 protein contains a Zinc-PHD finger domain, a bromodomain, a PWWP domain, and a MYND type of zinc finger (Figure 3, www.ensembl.org). It functions as a receptor for activated C-kinase (RACK) protein [13] but is also a cutaneous T-cell lymphoma-associated antigen (http://www.ncbi.nlm.nih.gov/gene/23613). The RELA protein is related to c-Rel and its oncogenic avian derivative v-Rel protein, contains an RHD domain (Figure 3), and forms complexes with the most abundant form NFKB1 of the transcription factor NF-kappa-B [14] (http://www.ncbi.nlm.nih.gov/gene/5970). NF-kappa-B plays a key role in inflammatory and innate immune responses and its role in oncogenesis is being investigated [15], [16]. NF-kappa-B was found to be constitutively activated in CD34+ AML cells but not in the normal CD34+ hematopoietic cells [17], [18]. Supershift experiments identified both NFKB1 and RELA proteins in the myeloid blast nucleous and the presence of NFKB1/RELA heterodimer as well as NFKB1 and RELA homodimers [17], [18]. Constitutive activation of NFKB in AML cells may be caused by various mechanisms such as alterated kinase activity, receptor overexpression autocrine cytokine production, and viral infections [19]. So far, no structural aberrations or locus amplification of *RELA* have been found [20]. The present study is therefore the first in which a rearrangement of *RELA* was detected and perhaps the *ZMYND8-RELA* fusion gene might be another novel mechanism of constitutive activation of NFKB in AML cells since the RELA gene is under control of the *ZMYND8* promoter.

The putative ZMYND8-RELA fusion protein contains the Zinc-PHD finger domain, a bromodomain, a PWWP domain, a MYND type of zinc finger of ZMYND8, and the entire RELA protein (Figure 3), indicating that it might influence several cellular processes including the NF-kappa-B pathway [15], [16]. Fusion genes caused by chromosomal aberrations often seen as the sole change at cytogenetic analysis are assumed to represent primary tumorigenic events, the paradigmatic example being *BCR-ABL* and the Philadelphia chromosome [8]. Thus, the combination of karyotyping by banding cytogenetics with next generation RNA sequencing is a powerful tool to detect such fusion genes in cancer as demonstrated both here and in our previous report [6].

The patient in this study presented initially with an MDS-like morphology, but evolved rapidly into a picture that morphologically resembled acute erythroid leukemia. The rapid increase in erythroid blasts was indicative of a true de novo AML (TDN-AML) biology. However, we cannot exclude the possibility that our patient may have evolved from MDS into an myelodysplasia-related AML (MDR-AML). The finding of a *ZMYND8/RELA* fusion as the central pathogenetic event does not shed more light on the early phenotypic features since no prior knowledge is at hand linking this fusion gene to any of the two said modes of disease presentation.
